# The Effect of COVID-19 on Herding Behavior in Eastern European Stock Markets

**DOI:** 10.3389/fpubh.2021.695931

**Published:** 2021-07-02

**Authors:** Hao Fang, Chien-Ping Chung, Yen-Hsien Lee, Xiaohan Yang

**Affiliations:** ^1^School of Economics, QuFu Normal University, Rizhao, China; ^2^Management Undergraduate Program, National Taiwan University of Science and Technology, Taipei, Taiwan; ^3^Department of Finance, Chung Yuan Christian University, Chungli, Taiwan; ^4^School of Economics, QuFu Normal University, Rizhao, China

**Keywords:** COVID-19, CSAD model, herding behavior, Eastern Europe, stock market, regional herding

## Abstract

Unlike past health crises that were more localized, the highly contagious coronavirus disease 2019 (COVID-19) crisis is impacting the world to an unprecedented extent. This is the first study examining how and whether the COVID-19 pandemic affects herding behavior in the Eastern European stock markets. Using samples from the stock markets of Russia, Poland, the Czech Republic, Hungary, Croatia, and Slovenia from January 1, 2010 to March 10, 2021, we demonstrate that the COVID-19 pandemic has increased herding behavior in all the sample stock markets. Our results show that the COVID-19 crisis reinforces the impact of global market returns on herding behavior in these specific stock markets. We find that COVID-19 strengthens the spillover effect of regional herding on herding behavior. Thus, financial authorities should monitor investors in the stock market to avoid the increase in herding behavior as well as the reinforcement of the global market returns and regional return dispersion on herding during the period of pandemic.

## Introduction

The highly contagious coronavirus disease 2019 (COVID-19) pandemic first occurred in Wuhan, China in December 2019 and spread rapidly worldwide. By February 18, 2021, 1.1 billion people in 218 countries were infected with the virus. Among them, there have been 2.44 million deaths globally and over 80 million recovered patients. In Eastern Europe, the first confirmed case appeared in Russia on January 31, 2020, and subsequent related cases gradually appeared and spread to other countries such as Croatia on February 25, 2020, Czech Republic on March 1, 2020, Poland and Hungary on March 4, 2020, and Slovenia on March 5, 2020 (see Table 1). The influence of COVID-19 is an unprecedented global disaster, different from the previous pandemic events that were more localized. Furthermore, the virus is resilient and is expected to produce a long-term destructive impact on world health and the economy.

**Table 1:** The information for our sample countries in Eastern Europe.

**Sr.no**.	**Country**	**The date of 1st COVID-19 confirmed case**	**Stock index**	**Active stock exchange day**
1	Russia	Jan 31, 2020	MOEX	2010/01/01
2	Poland	Mar 04, 2020	WIG 30	2009/01/30
3	Czech Republic	Mar 01 2020	PX	2009/08/30
4	Hungary	Mar 04, 2020	Budapest SEBudapest SE	2009/12/14
5	Croatia	Feb 25, 2020	CROBEX	2008/11/19
6	Slovenia	Mar 05, 2020	Blue-Chip SBITOP	2010/01/01

The past literature on the effects of pandemics on financial markets is limited. Among them, pandemics can affect financial systems through huge economic costs (1–3). Ali et al. (4) show empirically that the Chinese stock market exhibited relatively lower volatility during both the epidemic and pandemic period, while the average volatility of stock markets in the US, the UK, Germany, and South Korea increased drastically from the epidemic period to the pandemic period. So et al. (5) investigate the effects of the COVID-19 pandemic on the connectedness of stock returns in the Hong Kong market. Compared to other financial crises, there is a large increase in the network connectedness in financial networks during the COVID-19 outbreak. Al-Awadhi et al. (6) examine whether contagious diseases affect the stock returns of the Hang Seng Index and the Shanghai Stock Exchange Composite Index during the COVID-19 outbreak. Their results show that both the daily growth in total confirmed cases and total death caused by COVID-19 produce significantly negative effects on stock returns. Mazur et al. (7) examine the differential stock price reactions to the rapid spread of the coronavirus. Their results show that the stock prices in most sectors collapse, while others may benefit from the pandemic. Albulescu (8) examines the impact of official announcements regarding the new confirmed cases and fatality ratio on the volatility of US stock markets. Their results show that the contagion of the COVID-19 strengthens the S&P 500 stock volatility.

Specifically, pandemics can result in investor herding behaviors in the stock market. This issue requires further analysis because the official announcement of World Health Organization (WHO) categorizing the COVID-19 outbreak as a global pandemic has led to serious crashes in global stock markets. Herding behaviors in the stock market denote that investors face uncertain information, and thus, they tend to follow the stock-investing decisions and actions of others or depend too much on public information without heeding their own private information (9). Pandemics such as COVID-19 can raise investors' fears in stock return uncertainty due to medical and economic instability, and thus, investors can imitate the behavior of others who are more informed, which can cause investor herding behaviors in the stock market.

Moreover, there are few studies on herding behavior in the Eastern European stock markets. Among them, Filip et al. (10) use the cross-sectional absolute deviation (*CSAD*) model to investigate the existence of herding behavior in Eastern European stock markets. Their results show the evidence of herding behavior of investors at the sector level for most stock markets, except Poland. Furthermore, the herding behavior exists especially during the period of market downturn. By using the *CSAD* model, Angela-Maria et al. (11) examine the herding behavior for size-ranked stock portfolios in 10 Central Eastern European (CEE) stock markets. They find that herding is significant for the largest stock portfolios in Bulgaria, Slovenia, and Latvia, while herding exists for medium-sized portfolios in Estonia. Moreover, they find evidence on herding behavior during the financial crisis period.

There are some studies on the effects of pandemics in Eastern European countries that center on how pandemics affect public health and the environment. Kowalska et al. (12) examine the effect of the COVID-19 pandemic on HIV care and persistence of antiretroviral treatment (ART) supplies in Central and Eastern European (CEE) countries. This investigation is especially important because the fragile healthcare systems in these countries are tackling the COVID-19 pandemic. Their results show that the treatment for both severe and chronic medical situations unrelated to COVID-19 have been affected, and maintaining drug supplies has been difficult. Filonchyk et al. (13) examine the effect of COVID-19 prevention measures on air quality. They find that the restrictions aimed at preventing the spread of coronavirus improve the air quality in Poland. However, this impact can be limited because emissions increased after lockdown was eased due to the gradual increase in human activity. Morozova et al. (14) investigate the potential natural sources of plague in Europe. Their results reveal that black rats were at least one of the possible natural sources of plague spread in Eastern Europe.

Considering only a few studies focus either on herding behavior in Eastern European stock markets or on the effects of pandemics in Eastern European countries, it is worthwhile to combine the two areas of research to analyze the impacts of pandemics on herding behavior in Eastern European stock markets. The research objective of this study is to investigate whether COVID-19 enhances the existence of herding behavior, reinforces the effect of the global market returns on herding behavior, and strengthens the spillover effect of regional herding on herding behavior in Eastern European stock markets. Specifically, this study uses the *CSAD* model's extension to implement these issues. The *CSAD* model centers on the degree of dispersion of an investor for stocks' returns, which is regarded as the standard in the literature on the herding in stock market. Our empirical findings show that the COVID-19 pandemic has increased herding behavior in all Eastern European stock markets. The COVID-19 crisis reinforces the impact of global market returns on herding behavior in these specific stock markets. Moreover, COVID-19 strengthens the spillover effect of regional herding on herding behavior.

This is the first study examining how and whether the COVID-19 pandemic affects herding behavior in the Eastern European stock markets, which fills this gap. The remainder of this paper proceeds as follows. Section Materials and Methods illustrates the materials and methods, including data, the *CSAD* measure and its model, and the *CSAD* model's extension. Section Results and Discussions reports and discusses our empirical results. Section Conclusions concludes our paper.

## Materials and Methods

### Data

The main data in this study are collected from stock market indexes and individual stock prices of firms listed on MOEX (Russia), WIG 30 (Poland), PX (Czech Republic), Budapest SE (Hungary), CROBEX (Croatia), and Blue-Chip SBITOP (Slovenia). The individual stock prices of all firms listed on our six Eastern European stock markets are taken from Datastream dataset, which is developed by Thomson Company^1^. The daily stock price of these individual stocks was downloaded and transformed into a natural logarithm before the calculation of the daily stock returns. Our sample data also includes global stock prices, proxied by the MSCI stock index. Our sample period is from January 1, 2010 to March 10, 2021^2^. These stock markets are selected because they are the markets where there was enough trading information for constituent stocks of market index during the sample period and were impacted by COVID-19 in Eastern European countries^3^.

Our total sample includes 2,913 observations in the Moscow Stock Exchange (MSE), Warsaw Stock Exchange (WSE), Prague Stock Exchange (PSE), Budapest Stock Exchange (BSE), Zagreb Stock Exchange (ZSE), and Ljubljana Stock Exchange (LJSE), respectively. Because we use daily data to calculate investor herding behavior in the stock market, the daily stock returns are computed as *R*_*i,t*_ = (*P*_*i,t*_ – *P*_*i, t*−1_)/*P*_*i, t*−1_.

### CSAD Measure and Its Model

To examine investor herding behavior, this study first uses the return dispersion model proposed by Chang et al. (15), which is an improvement of the model proposed by Christie and Huang (16). The Lakonishok et al. (17) herding measure uses trading data based on the probabilities that follow the hypothesis of an efficient market, but it is not necessary for return dispersion herding model to follow this assumption since this model follows the CAPM setting. Meanwhile, the *LSV* measure needs the trading data with specific stock, specific time, and number of investors, while the return dispersion model only needs the trading data with specific stock and specific time. The return dispersion model that needs less data dimension is more likely to calculate and compare investor herding behavior in many countries than the *LSV* measure. Chang et al. (15) employs the *CSAD* of returns as a measure of return dispersion formulated as follows:

(1) $$\begin{array}{r} CSAD_{t}=\frac{1}{M}\overset{M}{\underset{i=1}{\sum }}|R_{i,t}-R_{m,t}| \end{array}$$

where *R*_*i,t*_ is the individual return of stock *i* on day *t* and *R*_*m,t*_ is the market return (equal-weighted average stock return) on day *t* in a country-specific stock market. Our *CSAD* model in the country-specific stock market is the following:

(2) $$\begin{array}{r} CSAD_{t}=\alpha +\gamma_{1}|R_{\;m,t}|+\gamma_{2}R_{m,t}^{2}+\varepsilon_{t} \end{array}$$

where the definitions of *R*_*m, t*_ and *CSAD*_*t*_ are the same as in Equation (1). γ_1_ is expected to be significantly positive because the cross-sectional dispersion of returns is supposed to increase with the level of absolute market returns. If herding exists, this relationship between *CSAD* and market return may become less positive and linear and might even turn negative and non-linear. This phenomenon occurs because herding denotes a decrease in *CSAD* and an increase in extreme market returns. Thus, herding is assumed to exhibit a significantly negative γ_2_.

### Empirical Examination of the CSAD Model's Extension

Then, we add the squared market returns of the global stock prices to examine if the global stock returns produce the impacts on investor herding behavior in the country-specific stock market. The test is shown in Equation (3) as follows:

(3) $$\begin{array}{r} CSAD_{t}=\alpha +\gamma_{1}|R_{\;m,t}|+\gamma_{2}R_{m,t}^{2}+\gamma_{3}R_{Gm,t}^{2}+\varepsilon_{t} \end{array}$$

where *R*_*Gm,t*_ is the returns of the global stock prices, which is proxied by the MSCI stock index returns. The significantly negative value of γ_3_ in Equation (3) indicates that the country-specific stock market exhibits herding around the global market returns.

Next, we add the regional return dispersions in the Eastern European countries to examine the existence of the regional herding spillover from the regional stock market to the country-specific stock market. The examination is given in Equation (4) as

(4) $$\begin{array}{r} CSAD_{t}=\alpha +\gamma_{1}|R_{m,t}|+\gamma_{2}R_{m,t}^{2}+\gamma_{3}R_{Gm,t}^{2}+\gamma_{4}CSAD_{t}^{L}+\varepsilon_{t} \end{array}$$

where $CSAD_{t}^{L}$ is the regional return dispersions in the East European stock markets. The significantly positive value of γ_4_ in Equation (4) means that the regional return dispersions spread out to raise the herding behavior of country-specific stock markets. The *CSAD* model's extension fits in this study since the global stock returns can affect herding behavior in the country-specific stock market, and the spillover effect of the regional herding on herding behavior can occur.

More importantly, we add the COVID-19 dummy variable (*D*^*COVID*^) to examine the effect of COVID-19 on investor herding in the regional stock markets. This is presented in Equation (5) as

(5) $$\begin{array}{l} CSAD_{t}=\alpha +\gamma_{11}D^{COVID}|R_{m,t}|+\gamma_{12}\left(1-D^{COVID}\right)|R_{m,t}|\\ \;+\gamma_{21}D^{COVID}R_{m,t}^{2}+\gamma_{22}\left(1-D^{COVID}\right)R_{m,t}^{2}\\ \;+\gamma_{31}D^{COVID}R_{Gm,t}^{2}+\gamma_{32}\left(1-D^{COVID}\right)R_{Gm,t}^{2}\\ \;+\gamma_{41}D^{COVID}CSAD_{t}^{L}+\gamma_{42}\left(1-D^{COVID}\right)CSAD_{t}^{L}+\varepsilon_{t} \end{array}$$

where *D*^*COVID*^ is the dummy variable measuring whether COVID-19 occurs. *D*^*COVID*^ = 1 since the date of the first confirmed COVID-19 case in each sample country (see Table 1) and zero before that. The significantly negative values of γ_21_ and γ_22_ in Equation (5) denote the existence of herding following (before) COVID-19. Similarly, the significantly negative values of γ_31_ and γ_32_ indicate that country-specific stock markets exhibit herding around the global market returns following (before) COVID-19. Moreover, the significantly positive values of γ_41_ and γ_42_ imply that the herding of country-specific stock markets increases by the regional herding spillover following (before) COVID-19.

In addition, we take the previous effect of COVID-19 on stock herding in Equation (5) as our general model to further consider the asymmetric effects that can affect herding behavior: up- and down-market returns (18–20). This study only considers the asymmetry of market returns, since the rise and fall of stock market returns are the most intuitive form of market asymmetry for investors. Specifically, we investigate whether the asymmetry of herding behavior exists when the stock market is rising or falling during the pre- and post-COVID-19 periods.

## Results and Discussions

Table 2 reports the descriptive statistics for *CSAD* measure, the average market return (*R*_*m,t*_) calculated using the equal weights for each sample markets, the global market return (*R*_*Gm,t*_) proxied by the MSCI index return, and the regional *CSAD* measure calculated using the regional return dispersions ($CSAD_{t}^{L}$). We can see from Table 2 that the mean values and standard deviations of *CSAD* in all sample stock markets and *CSAD*^*L*^ are higher during the COVID-19 period. Our results denote that there are unusual cross-sectional variations because of unexpected events (21, 22). Meanwhile, the mean values of both the specific and global market returns are lower, and their standard deviations are higher during the COVID-19 period. They suggest that COVID-19 has caused a significant reduction and instability in the profitability of the stock markets both in Eastern Europe and the broader world.

**Table 2:** Descriptive statistics.

		**N^**°**^ Obs**.	**Mean**	**Std.Dev**.	**Min**	**Max**
**MSE** (Russia)	*CSAD*	2651	0.018	0.014	0.000	0.248
Pre COVID-19	*R*_*M,t*_	2651	0.000	0.016	0.142	0.123
	*R*_*GM,t*_	2647	0.000	0.008	0.053	0.048
	$CSAD_{t}^{L}$	2650	0.006	0.004	0.000	0.090
**MSE** (Russia)	*CSAD*	268	0.019	0.016	0.000	0.140
Post COVID-19	*R*_*M,t*_	268	0.001	0.021	0.135	0.137
	*R*_*GM,t*_	283	0.000	0.018	0.104	0.084
	$CSAD_{t}^{L}$	268	0.007	0.005	0.000	0.032
**WSE** (Poland)	*CSAD*	2651	0.012	0.004	0.000	0.040
Pre COVID-19	*R*_*M,t*_	2651	0.000	0.010	0.062	0.044
	*R*_*GM,t*_	2647	0.000	0.008	0.053	0.048
	$CSAD_{t}^{L}$	2650	0.006	0.004	0.000	0.090
**WSE** (Poland)	*CSAD*	268	0.019	0.009	0.000	0.070
Post COVID-19	*R*_*M,t*_	268	0.001	0.026	0.151	0.078
	*R*_*GM,t*_	283	0.000	0.018	0.104	0.084
	$CSAD_{t}^{L}$	268	0.007	0.005	0.000	0.032
**PSE** (Czech)	*CSAD*	2651	0.009	0.006	0.000	0.122
Pre COVID-19	*R*_*M,t*_	2651	0.000	0.009	0.048	0.071
	*R*_*GM,t*_	2647	0.000	0.008	0.053	0.048
	$CSAD_{t}^{L}$	2650	0.006	0.004	0.000	0.090
**PSE** (Czech)	*CSAD*	268	0.019	0.006	0.000	0.042
Post COVID-19	*R*_*M,t*_	268	0.001	0.014	0.083	0.061
	[[Inline Image]]	283	0.000	0.018	0.104	0.084
	$CSAD_{t}^{L}$	268	0.007	0.005	0.000	0.032
**BSE** (Hungary)	*CSAD*	2651	0.022	0.017	0.000	0.319
Pre COVID-19	*R*_*M,t*_	2651	0.000	0.014	0.185	0.175
	*R*_*GM,t*_	2647	0.000	0.008	0.053	0.048
	$CSAD_{t}^{L}$	2650	0.006	0.004	0.000	0.090
**BSE** (Hungary)	*CSAD*	268	0.028	0.018	0.000	0.076
Post COVID-19	*R*_*M,t*_	268	0.000	0.020	0.139	0.075
	*R*_*GM,t*_	283	0.000	0.018	0.104	0.084
	$CSAD_{t}^{L}$	268	0.007	0.005	0.000	0.032
**ZSE** (Croatia)	*CSAD*	2651	0.015	0.012	0.000	0.331
Pre COVID-19	*R*_*M,t*_	2651	0.001	0.011	0.208	0.065
	*R*_*GM,t*_	2647	0.000	0.008	0.053	0.048
	$CSAD_{t}^{L}$	2650	0.006	0.004	0.000	0.090
**ZSE** (Croatia)	*CSAD*	268	0.016	0.019	0.000	0.058
Post COVID-19	*R*_*M,t*_	268	0.000	0.012	0.091	0.060
	*R*_*GM, t*_	283	0.000	0.018	0.104	0.084
	$CSAD_{t}^{L}$	268	0.007	0.005	0.000	0.032
**LJSE** (Slovenia)	*CSAD*	2651	0.014	0.011	0.000	0.191
Pre COVID-19	*R*_*M, t*_	2651	0.000	0.011	0.086	0.113
	*R*_*GM, t*_	2647	0.000	0.008	0.053	0.048
	$CSAD_{t}^{L}$	2650	0.006	0.004	0.000	0.090
**LJSE** (Slovenia)	*CSAD*	268	0.017	0.020	0.000	0.060
Post COVID-19	*R*_*M, t*_	268	0.000	0.013	0.080	0.048
	*R*_*GM, t*_	283	0.000	0.018	0.104	0.084
	$CSAD_{t}^{L}$	268	0.007	0.005	0.000	0.032

Table 3 shows the results of estimating Equation (5) for the general model in these Eastern European stock markets during our sample period. Our results show the significantly negative coefficient γ_21_ for most sample countries, except that the non-significant coefficient γ_21_ in the Warsaw Stock Exchange of Poland is still negative. The findings mean that significant investor herding behavior exists in almost all the Eastern European stock markets. The only exception, the stock market of Poland, still shows a tendency for herding. Compared with γ_22_, γ_21_ in all the countries are more significantly negative, and the differences between γ_21_ and γ_22_ in all the countries are evident. The results denote that COVID-19 enhances investor herding behavior in all the Eastern European stock markets. This phenomenon possibly occurs because COVID-19 causes investors' fears in stock return uncertainty, and investors easily follow the trading behavior of others in the stock market.

**Table 3:** Effect of COVID-19 on herding behaviour in Eastern Europe markets for the general model.

**Panel A:MSE (Russia)**						
γ_11_	γ_12_	γ_21_	γ_22_	γ_31_	γ_32_	
0.061	−0.001	−6.138^***^	9.258^***^	−2.389^*^	5.362^***^	
[0.177]	[0.962]	[0.000]	[0.000]	[0.098]	[0.005]	
γ_41_	γ_42_	α	Obs.	R-squared	t-stat1 (H0:γ_21_ = γ_22_)	t-stat2 (H0:γ_31_ = γ_32_)
1.145^***^	0.829^***^	0.010^***^	2913	0.516	172.38^***^	13.50^*^
[0.000]	[0.000]	[0.000]				
**Panel B:WSE (Poland)**						
γ_11_	γ_12_	γ_21_	γ_22_	γ_31_	γ_32_	
0.118^***^	0.039^***^	−1.323	6.925^***^	−1.035	−0.436	
[0.000]	[0.000]	[0.133]	[0.000]	[0.561]	[0.614]	
γ_41_	γ_42_	α	Obs.	R-squared	t-stat1 (H0:γ_21_ = γ_22_)	t-stat2 (H0:γ_31_ = γ_32_)
0.808^***^	0.082^***^	0.011^***^	2913	0.328	10.324^*^	5.251
[0.000]	[0.002]	[0.000]				
**Panel C:PSE (Czech)**						
γ_11_	γ_12_	γ_21_	γ_22_	γ_31_	γ_32_	
0.057^*^	0.016	−6.386^***^	15.923^***^	−3.033^**^	0.617	
[0.092]	[0.540]	[0.004]	[0.000]	[0.011]	[0.729]	
γ_41_	γ_42_	α	Obs.	R-squared	t-stat1 (H0:γ_21_ = γ_22_)	t-stat2 (H0:γ_31_ = γ_32_)
0.477^***^	0.057	0.008^***^	2913	0.362	61.75^**^	102.52^***^
[0.000]	[0.251]	[0.000]				
**Panel D:BSE (Hungary)**						
γ_11_	γ_12_	γ_21_	γ_22_	γ_31_	γ_32_	
0.144^***^	0.067	−5.043^*^	11.492^***^	−1.068	−0.648	
[0.007]	[0.133]	[0.080]	[0.000]	[0.443]	[0.678]	
γ_41_	γ_42_	α	Obs.	R-squared	t-stat1 (H0:γ_21_ = γ_22_)	t-stat2 (H0:γ_31_ = γ_32_)
0.160	0.434^*^	0.017^***^	2913	0.578	14.28^*^	1.51
[0.293]	[0.061]	[0.000]				
**Panel E:ZSE (Croatia)**						
γ_11_	γ_12_	γ_21_	γ_22_	γ_31_	γ_32_	
0.111	0.083	−7.351^**^	8.411^***^	−1.657^*^	−1.125	
[0.108]	[0.148]	[0.021]	[0.000]	[0.081]	[0.232]	
γ_41_	γ_42_	α	Obs.	R-squared	t-stat1 (H0:γ_21_ = γ_22_)	t-stat2 (H0:γ_31_ = γ_32_)
0.243^***^	0.182^**^	0.013^***^	2913	0.611	62.56^**^	24.88^*^
[0.004]	[0.010]	[0.000]				
**Panel F:LJSE (Slovenia)**						
γ_11_	γ_12_	γ_21_	γ_22_	γ_31_	γ_32_	
0.037	−0.035	−10.533^***^	21.700^***^	−2.234^**^	−1.172	
[0.680]	[0.397]	[0.000]	[0.000]	[0.046]	[0.220]	
γ_41_	γ_42_	α	Obs.	R-squared	t-stat1 (H0:γ_21_ = γ_22_)	t-stat2 (H0:γ_31_ = γ_32_)
0.155^**^	0.147^**^	0.011^***^	2913	0.506	114.08^***^	18.27^*^
[0.020]	[0.023]	[0.000]				

*The t-stat1 (H0:γ_21_ = γ_22_) denotes the t-statistic examination for the difference between herding before COVID-19 and herding following COVID-19. The t-stat2 (H0:γ_31_ = γ_32_) denotes the t-statistic examination for the difference between herding caused by the global market returns before COVID-19 and that following COVID-19. p-values in brackets*.

* *p < 0.1*,

** *p < 0.05*,

*** *p < 0.01*.

Moreover, there is the significantly negative coefficient γ_31_ in the stock markets of most countries except the non-significantly negative γ_31_ in those of Poland and Hungary. This finding indicates that global stock returns reinforce investor herding behavior in the specific stock market of almost all the sample countries. Similarly, γ_31_ in most countries is more significantly negative than γ_32_. The findings provide evidence that COVID-19 strengthens the herding of country-specific stock markets in most Eastern European countries around the global market returns. Next, the significantly positive coefficient γ_41_ exists in the stock markets of most countries except the non-significantly positive γ_41_ in the stock market of Hungary. This phenomenon denotes that the regional return dispersions in Eastern Europe spread out to reinforce the herding behavior of country-specific stock markets. Additionally, γ_41_ in most countries are more significantly positive than γ_42_. The results imply that COVID-19 enhances the spillover of regional herding on the herding of country-specific stock markets in most Eastern European countries.

Moreover, Table 4 presents the results of considering the asymmetric effects of market return in each stock market. Compared with the results of the general model, those considering the asymmetric effects are robust in examining the coefficients affecting the *CSAD* measure in almost all Eastern European stock markets. Overall, regardless of higher or lower market returns, γ_21_ is more significantly negative than γ_22_, γ_31_ is more significantly negative than γ_32_, and γ_41_ is more significantly positive than γ_42_ in most of the sample countries. Accordingly, we reconfirm that COVID-19 raises herding behavior, reinforces country-specific stock market herding around the global market returns, and enhances the spillover of the regional herding on country-specific stock market in most Eastern European countries. In addition, herding behavior is more pronounced when market returns are lower during the post-COVID-19 period in the stock markets of most countries except Hungary and Russia.

**Table 4:** Effect of COVID-19 on herding behaviour in Eastern Europe markets for asymmetric effects.

**Panel A:MSE (Russia)**						
*R*_m, t_>0						
γ_11_	γ_12_	γ_21_	γ_22_	γ_31_	γ_32_	
0.372^***^	0.491^***^	−3.985^***^	3.273^***^	−2.879^***^	8.280^***^	
[0.000]	[0.000]	[0.000]	[0.000]	[0.006]	[0.001]	
γ_41_	γ_42_	α	Obs.	R-squared	t-stat1 (H0:γ_21_ = γ_22_)	t-stat2 (H0:γ_31_ = γ_32_)
0.853^***^	0.797^***^	0.007^***^	1444	0.556	120.71^***^	95.88^***^
[0.000]	[0.000]	[0.000]				
*R*_m, t_ <0						
γ_11_	γ_12_	γ_21_	γ_22_	γ_31_	γ_32_	
−0.578^***^	−0.552^***^	−1.892^*^	2.955^***^	−3.656^***^	1.393	
[0.000]	[0.000]	[0.055]	[0.000]	[0.001]	[0.607]	
γ_41_	γ_42_	α	Obs.	R-squared	t-stat1 (H0:γ_21_ = γ_22_)	t-stat2 (H0:γ_31_ = γ_32_)
1.041^***^	0.659^***^	0.008^***^	1328	0.590	49.22^**^	78.45^**^
[0.000]	[0.010]	[0.000]				
**Panel B:WSE (Poland)**						
*R*_m, t_>0						
γ_11_	γ_12_	γ_21_	γ_22_	γ_31_	γ_32_	
0.228^***^	0.128^**^	−2.350^*^	3.061	−0.670	1.764^*^	
[0.003]	[0.016]	[0.052]	[0.191]	[0.353]	[0.088]	
γ_41_	γ_42_	α	Obs.	R-squared	t-stat1 (H0:γ_21_ = γ_22_)	t-stat2 (H0:γ_31_ = γ_32_)
0.511^***^	−0.026	0.012^***^	1445	0.457	24.08^*^	8.205^*^
[0.000]	[0.331]	[0.000]				
*R*_m, t_ <0						
γ_11_	γ_12_	γ_21_	γ_22_	γ_31_	γ_32_	
−0.209^***^	−0.105^***^	−2.497^***^	2.561^***^	−0.838	0.360	
[0.001]	[0.000]	[0.000]	[0.004]	[0.105]	[0.728]	
γ_41_	γ_42_	α	Obs.	R-squared	t-stat1 (H0:γ_21_ = γ_22_)	t-stat2 (H0:γ_31_ = γ_32_)
0.372^**^	0.012	0.012^***^	1345	0.442	186.510^***^	4.112
[0.011]	[0.586]	[0.000]				
**Panel C:PSE (Czech)**						
*R*_m, t_>0						
γ_11_	γ_12_	γ_21_	γ_22_	γ_31_	γ_32_	
0.344^***^	0.086	−1.486^*^	15.314^***^	−2.052^*^	1.699	
[0.004]	[0.394]	[0.046]	[0.004]	[0.084]	[0.520]	
γ_41_	γ_42_	α	Obs.	R-squared	t-stat1 (H0:γ_21_ = γ_22_)	t-stat2 (H0:γ_31_ = γ_32_)
−0.074	0.183^*^	0.008^***^	1461	0.441	32.52^**^	88.07^**^
[0.224]	[0.094]	[0.000]				
*R*_m, t_ <0						
γ_11_	γ_12_	γ_21_	γ_22_	γ_31_	γ_32_	
−0.338^***^	−0.258^**^	−2.142^*^	6.909	−2.057^*^	0.449	
[0.002]	[0.012]	[0.057]	[0.222]	[0.0824]	[0.834]	
γ_41_	γ_42_	α	Obs.	R-squared	t-stat1 (H0:γ_21_ = γ_22_)	t-stat2 (H0:γ_31_ = γ_32_)
0.253^*^	0.035	0.007^***^	1338	0.339	13.58^*^	92.03^**^
[0.075]	[0.527]	[0.000]				
**Panel D:BSE (Hungary)**						
*R*_m, t_>0						
γ_11_	γ_12_	γ_21_	γ_22_	γ_31_	γ_32_	
0.656^***^	0.835^***^	−4.994^***^	5.422^***^	−2.654	−2.561	
[0.000]	[0.000]	[0.000]	[0.000]	[0.182]	[0.384]	
γ_41_	γ_42_	α	Obs.	R-squared	t-stat1 (H0:γ_21_ = γ_22_)	t-stat2 (H0:γ_31_ = γ_32_)
0.038	0.042	0.015^***^	1390	0.698	240.34^***^	5.148
[0.766]	[0.641]	[0.000]				
*R*_m, t_ <0						
γ_11_	γ_12_	γ_21_	γ_22_	γ_31_	γ_32_	
−0.064	−0.778^***^	−4.260^***^	5.405^***^	−2.960^**^	−2.235	
[0.440]	[0.000]	[0.000]	[0.000]	[0.016]	[0.283]	
γ_41_	γ_42_	α	Obs.	R-squared	t-stat1 (H0:γ_21_ = γ_22_)	t-stat2 (H0:γ_31_ = γ_32_)
0.233	0.062	0.014^***^	1393	0.731	208.12^***^	12.25^*^
[0.130]	[0.313]	[0.000]				
**Panel E:ZSE (Croatia)**						
*R*_m, t_>0						
γ_11_	γ_12_	γ_21_	γ_22_	γ_31_	γ_32_	
0.677^***^	0.233^***^	−2.411^*^	22.386^***^	−2.367^*^	−2.325^*^	
[0.000]	[0.003]	[0.081]	[0.000]	[0.073]	[0.082]	
γ_41_	γ_42_	α	Obs.	R-squared	t-stat1 (H0:γ_21_ = γ_22_)	t-stat2 (H0:γ_31_ = γ_32_)
0.001	0.006	0.013^***^	1354	0.568	60.85^**^	2.42
[0.985]	[0.963]	[0.000]				
*R*_m, t_ <0						
γ_11_	γ_12_	γ_21_	γ_22_	γ_31_	γ_32_	
−0.896^***^	−0.651^***^	−4.582^***^	4.233^***^	−6.678^**^	−6.052^*^	
[0.000]	[0.000]	[0.000]	[0.000]	[0.021]	[0.061]	
γ_41_	γ_42_	α	Obs.	R-squared	t-stat1 (H0:γ_21_ = γ_22_)	t-stat2 (H0:γ_31_ = γ_32_)
0.090	−0.067^*^	0.011^***^	1430	0.830	235.15^***^	22.23^*^
[0.488]	[0.071]	[0.000]				
**Panel F:LJSE (Slovenia)**						
*R*_m, t_>0						
γ_11_	γ_12_	γ_21_	γ_22_	γ_31_	γ_32_	
0.797^***^	0.742^***^	−3.195^*^	8.551^***^	−1.452^**^	0.830	
[0.000]	[0.000]	[0.076]	[0.000]	[0.040]	[0.530]	
γ_41_	γ_42_	α	Obs.	R-squared	t-stat1 (H0:γ_21_ = γ_22_)	t-stat2 (H0:γ_31_ = γ_32_)
0.368	−0.010	0.008^***^	1388	0.651	89.54^**^	50.88^**^
[0.114]	[0.821]	[0.000]				
*R*_m, t_ <0						
γ_11_	γ_12_	γ_21_	γ_22_	γ_31_	γ_32_	
−1.248^***^	−0.748^***^	−6.076^***^	10.674^***^	−1.784^***^	−1.437^*^	
[0.000]	[0.000]	[0.000]	[0.000]	[0.007]	[0.070]	
γ_41_	γ_42_	α	Obs.	R-squared	t-stat1 (H0:γ_21_ = γ_22_)	t-stat2 (H0:γ_31_ = γ_32_)
0.546^*^	−0.108^*^	0.009^***^	1389	0.628	108.07^***^	11.69^*^
[0.061]	[0.054]	[0.000]				

*The t-stat1 (H0:γ_21_ = γ_22_) denotes the t-statistic examination for the difference between herding before COVID-19 and herding following COVID-19. The t-stat2 (H0:γ_31_ = γ_32_) denotes the t-statistic examination for the difference between herding caused by the global market returns before COVID-19 and that following COVID-19. p-values in brackets*.

* *p < 0.1*,

** *p < 0.05*,

*** *p < 0.01*.

## Conclusions

COVID-19 has affected the world in multiple ways. However, few studies have explored the effects of such a pandemic on herding behavior in stock markets. Importantly, there is no research until now that analyzes the related issues in Eastern European countries.

This study first examines whether the COVID-19 pandemic influences herding behavior in Eastern European stock markets. Using the samples of the stock prices of firms listed on MOEX (Russia), WIG 30 (Poland), PX (Czech Republic), Budapest SE (Hungary), CROBEX (Croatia), and Blue-Chip SBITOP (Slovenia) from January 1, 2010 to March 10, 2021, we demonstrate that the COVID-19 pandemic raises herding behavior in all Eastern European stock markets.

This study then investigates whether the global stock market affects herding in country-specific stock markets since the occurrence of COVID-19. Our results show that the COVID-19 crisis reinforces the impact of the global market returns on herding behavior of specific stock markets in most Eastern European countries. Next, we investigate whether the regional return dispersions spread out to affect herding in the specific stock markets since the COVID-19 crisis. We find that COVID-19 strengthens the spillover effect of the regional herding on herding behavior of specific stock markets in almost all the Eastern European countries.

Finally, we examine the asymmetric effects of herding behavior by considering up-/down-market returns during the periods pre- and post-COVID-19. Our results on the asymmetric effects are still robust for all the above tests in most Eastern European countries. Considering the asymmetric effects, we reconfirm that COVID-19 increases herding behavior, reinforces the effect of the global market returns on herding behavior, and strengthens the spillover effect of regional herding on herding behavior in these countries. Accordingly, financial authorities should monitor investor behavior in the stock market to avoid the increase in their herding behavior as well as the reinforcement of the global market returns and regional return dispersion on herding during the pandemic period. Especially, financial authorities in developing countries should formulate policies quickly to reduce investors' fears in their health and the economy as a pandemic occurs. Because medical resources and financial developments are limited in developing countries, investors in these countries are more likely to follow the trading behavior of others in the stock market due to their fears in such a pandemic. Meanwhile, because the stock prices in developing countries are easily affected by the global stock indices and the regional stock dispersions, financial authorities in these countries should formulate policies fast to decrease the reinforcement of the global market returns and the regional stock herding on herding behavior in stock markets of these countries as facing a pandemic.

The limitation of this study is that several stock markets in Eastern European countries are not selected in our sample countries. The reason is because there was not enough trading information for constituent stocks of the market index in these markets during our sample period. In the further research, researches could consider the following issues. First, by adding the global return dispersions into CSAD model, they can investigate the evidence of the global herding spillover from the global stock market to the country-specific stock market since the pandemic occurs. Second, they can examine the effects of the different stages of the pandemic on herding behavior in stock markets.

## Data Availability Statement

The original contributions presented in the study are included in the article/supplementary material, further inquiries can be directed to the corresponding author/s.

## Author Contributions

HF: writing this manuscript. C-PC: providing datasets and discussing. Y-HL: running program. XY: finding literatures. All authors contributed to the article and approved the submitted version.

## Conflict of Interest

The authors declare that the research was conducted in the absence of any commercial or financial relationships that could be construed as a potential conflict of interest.

## Footnotes

^1^The Thomson company is located in Stamford, CT, USA.

^2^This study considers January 1, 2010 as the initial date of our sample period because we avoid the harmful impact of the subprime financial crisis period, and most stock markets in Eastern European countries became active after the financial crisis.

^3^The date of the first COVID-19 confirmed case in these countries are shown in Table 1.
